# The synaptic maintenance problem: membrane recycling, Ca^2+^ homeostasis and late onset degeneration

**DOI:** 10.1186/1750-1326-8-23

**Published:** 2013-07-08

**Authors:** Ilya Bezprozvanny, Peter Robin Hiesinger

**Affiliations:** 1 Department of Physiology, UT Southwestern Medical Center, 6001 Forest Park Road, Dallas 75390-9040, TX, USA; 2 Laboratory of Molecular Neurodegeneration, St Petersburg State Polytechnical University, 195251, St Petersburg, Russia

**Keywords:** Neurodegeneration, Endosome, Autophagy, Alzheimer’s disease, Calcium, Presenilin, Amyloid, Huntington’s disease, Hereditary motor and sensory neuropathy, Lysosomal storage disorder, Ataxia, Calcineurin, Excitotoxicity

## Abstract

Most neurons are born with the potential to live for the entire lifespan of the organism. In addition, neurons are highly polarized cells with often long axons, extensively branched dendritic trees and many synaptic contacts. Longevity together with morphological complexity results in a formidable challenge to maintain synapses healthy and functional. This challenge is often evoked to explain adult-onset degeneration in numerous neurodegenerative disorders that result from otherwise divergent causes. However, comparably little is known about the basic cell biological mechanisms that keep normal synapses alive and functional in the first place. How the basic maintenance mechanisms are related to slow adult-onset degeneration in different diseasesis largely unclear. In this review we focus on two basic and interconnected cell biological mechanisms that are required for synaptic maintenance: endomembrane recycling and calcium (Ca^2+^) homeostasis. We propose that subtle defects in these homeostatic processes can lead to late onset synaptic degeneration. Moreover, the same basic mechanisms are hijacked, impaired or overstimulated in numerous neurodegenerative disorders. Understanding the pathogenesis of these disorders requires an understanding of both the initial cause of the disease and the on-going changes in basic maintenance mechanisms. Here we discuss the mechanisms that keep synapses functional over long periods of time with the emphasis on their role in slow adult-onset neurodegeneration.

## Introduction

Proteins and organelles in all cells can become dysfunctional over time. Organisms utilize a variety of mechanisms to maintain cellular function and organ integrity. A straight-forward way to avoid intracellular maintenance problems is fast turnover of entire cells. Indeed, many cell types in the human body undergo turnover at rates that reflect their usage and exposure to harmful external or internal factors. For example, normal human liver cells have a turnover time of 1–2 years, red blood cells 4 months and skin epidermal cells undergo turnover on the scale of days [1,2]. Cellular turnover is one principle mechanism that can reduce the need to recognize, repair or remove dysfunctional proteins and organelles. However, the faster the cellular turnover, the higher the energy and resource costs. In addition, cellular morphology and tissue embedding render the turnover of some cell types difficult. Neurons are amongst the longest living cells in animals and largely exempt from cellular turnover. Regeneration of entire brains within days can be observed in the planarian flatworm [3]. However, such regenerative capabilities are rare in animals and typically affect entire body parts, rather than the replacement of individual cells inside morphologically complex tissues. The majority of neurons in the central nervous system from flies to men are long-lived cells that, once gone, are never replaced. This is at least partly due to the difficulty in re-wiring an individual neuron within the complicated network of the brain [4,5]. The precise placement of an individual cell in the skin or liver requires less information than the embedding of a pyramidal cell in the hippocampus or a dopaminergic neuron in the substantia nigra. Hence the complicated neuronal architecture highlights the necessity to keep individual neuron alive. In addition neurons store information about activity strength and plasticity in both pre- and postsynaptic nerve endings that is almost certainly lost if the cell is removed or replaced. Indeed, many synapses can, at least in theory, remain functional for the entire lifespan of an organism. A single neuron can support large numbers of synapses that are morphologically separated by long axonal and dendritic distances. Consequently, individual synapses regulate some aspects of their function and maintenance largely independently from each other and the cell body [6]. These properties set the stage for a unique maintenance problem at neuronal synapses.

The synaptic maintenance problem is further exacerbated by the tightly regulated high membrane turnover required for chemical neurotransmission. In the presynaptic terminal large numbers of synaptic vesicles undergo highly coordinated and Ca^2+^-regulated cycles of fusion and fission, endosomal sorting and renewal of their protein complement. Little is known about the rates at which vesicles and other organelles or individual proteins become dysfunctional during the function of the synapse. However, it is clear that dysfunctional vesicles, endosomes or endoplasmic reticulum may leak protons, Ca^2+^ and many other harmful ions and proteins. How failed vesicles or other compartments are recognized, sorted, degraded or repaired is only poorly understood. Over long periods of time even small defects in any of these basic physiological processes may lead to a slow reduction in neuronal function and health. In particular, endomembrane turnover and Ca^2+^ homeostasis play key roles for prolonged healthy neuronal and synaptic function [5,7,8]. Endomembrane turnover and Ca^2+^ cross-regulate each other and recent advances have highlighted examples how their concerted dysregulation underlies neuronal dysfunction [9-14]. Importantly, both represent homeostatic systems that, when mildly disrupted or otherwise functioning imperfectly, have been shown to lead to slow, adult-onset neurodegeneration [5,7]. In summary, even in the absence of any aberrant neurotoxic insult, the maintenance of the healthy function of neurons and synapses over decades is an astonishing biological feat. To what extent slow neurodegeneration, as is observed in numerous degenerative disorders, is causally linked to primary or secondary effects on neuronal maintenance mechanisms is the topic of this review.

### The maintenance problem of normal synapses

Similar to the possibility to replace entire cells, neurons have the option to replace entire synapses. Only few studies have explored such a ‘synaptic turnover’ mechanism. Long-term imaging studies in the barrel cortex of mice revealed that dendritic spines are actively eliminated in a sensory input-dependent manner during the animal’s lifespan. Loss of sensory input leads to reduced spine elimination [15]. Similarly, learning-dependent synapse formation, elimination and maintenance are tightly regulated by activity oscillations [16]. These observations are indications of an activity-dependent turnover mechanism [15]. Aberrant dendritic spine turnover may partly underlie lissencephaly in humans due to mutations in LIS1 [17]. Interestingly, the aging wild type mouse cortex is characterized by increased rates of axon terminal formation, elimination and destabilization. These findings are based on recent long-term multiphoton imaging results and indicate that there may be up to 20-fold higher synaptic turnover in an old versus young mouse brain, providing a possible explanation for late memory defects [18]. At neuromuscular junctions (NMJs) the removal and addition of presynaptic boutons is a common mode of strengthening, weakening and renewing the synapse [19,20]. However, individual bouton stability has been observed for long periods of time. The removal and addition of new boutons at the same NMJ is facilitated by the fact that every individual bouton has the same ‘synaptic specificity’ and the postsynaptic target cell is large. In contrast, at central synapses the specificity of pre- and postsynaptic partner pairings may often preclude synapse replacement. Indeed, individual central synapses have been shown to persist for long periods [21]. We are not aware of evidence that central synapses have an inherently limited lifetime, endowing them with the theoretical property to function throughout the life of the organism.

If turnover of entire neurons or synapses is not practical, then improved intracellular maintenance mechanisms are required [4,5,22]. At least two conceptually different approaches are available to the neuron: Individual proteins or organelles that have become dysfunctional may be individually recognized and repaired or degraded. Alternatively, proteins and organelles may have ‘built-in’ lifetimes that ensure that most proteins and organelles are functional at any given time, and no dysfunctional proteins or organelles accumulate. This maintenance mode allows for a minimum average functionality for all proteins or organelles of a certain type without the need to distinguish whether an individual protein or organelle has become dysfunctional. Examples for both maintenance modes have been characterized at synapses. Endomembrane degradation and Ca^2+^ homeostasis are two basic cellular mechanisms that are required for prolonged synaptic maintenance; defects in either mechanism ultimately leads to dysregulation of the other and is sufficient to cause slow adult-onset neurodegeneration [5].

**Figure 1:**
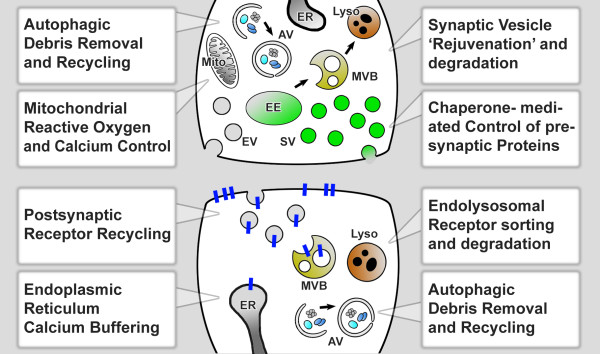
**Synaptic maintenance mechanisms.** Several basic maintenance mechanisms operate both pre- and postsynaptically to keep the synape healthy and functional over long periods of time. For each of the indicated processes disruptions have been shown to lead to premature synaptic degeneration independent of any specific neurotoxic insult or neurodegenerative disorder. ER - endoplasmic reticulum; AV - autophagic vacuole; Mito - Mitochondrium; EE - early endosome; EV - endocytic vesicle; SV - synaptic vesicle; Lyso - lysosome.

Little is known about the lifetime and degradation of synaptic vesicles [23]. Recent findings at the *Drosophila* neuromuscular junction suggest that synaptic vesicles are ‘rejuvenated’ through sorting at synaptic endosomal compartments [24]. The synaptic vesicle SNARE protein neuronal Synaptobrevin (n-Syb) plays a major role in both synaptic vesicle exocytosis and endolysosomal degradation at synapses [25]. However, it remains unclear when and how many synaptic vesicles cycle through the fusion with endosomal compartments (Figure 1). The target membrane SNARE protein SNAP25 is a direct target for the synaptic chaperone Cystein String Protein (CSP). Loss of CSP results in aberrant SNAP25 and defective SNARE complex formation [6] which causes degeneration in both flies and humans [26,27]. Defective CSP-dependent chaperoning increases degradation through the ubiquitin/proteasomal system (UPS). Similarly, the synaptic vesicle protein Synaptophysin undergoes degradation mediated by E3-ubiquitin protein ligases that are partly localized to endosomes [28]. Ubiquitination and proteosomal degradation play a major role in the maintenance of all cells. Defects in the UPS can lead to intracellular accumulation, which in turn trigger autophagy [29,30]. ESCRT proteins regulate the sorting of ubiquitinated cargo into multivesicular bodies [24,31]. Hence, defects in the UPS ultimately represent a challenge for endomembrane degradation at synapses. The precise roles and mechanisms of ubiquitination in neuronal maintenance are discussed elsewhere [6,32].

On the postsynaptic side, receptor cycling undergoes endolysosomal sorting, recycling and degradation steps that are reviewed elsewhere. Defects in these mechanisms can lead to dysfunctional synapses. However, comparably little is known about the roles of these maintenance mechanisms in relation to slow degeneration over long time periods [33,34]. In contrast, sustained Ca^2+^ -dependent signaling underlying synaptic plasticity provides clear leads for a role of Ca^2+^ homeostasis in postsynaptic maintenance. In the following sections on endomembrane degradation and Ca^2+^ homeostasis we therefore emphasize presynaptic mechanisms for endomembrane degradation and postsynaptic mechanisms in the case of Ca^2+^ homeostasis.

### Synaptic maintenance and endomembrane degradation

Failure to provide adequate quality control and degradation of pre- or post-synaptic trafficking compartments leads to the accumulation of dysfunctional intracellular machinery [5,35,36]. As the brain ages, changes in lipid composition accompany synaptic dysfunction and cognitive decline [37]. Furthermore, intracellular protein degradation decreases in aging neurons as compensatory endomembrane degradation increases. Hence, manipulation of endomembrane degradation has been suggested to decrease synaptopathogenesis associated with cognitive decline of the aging brain [38]. Several endomembrane degradation mechanisms operate at synapses, including autophagy, ubiquitous endolysosomal degradation and neuron-specific endolysosomal degradation. Defects in any of these mechanisms can lead to slow adult-onset neurodegeneration [5,39,40].

Autophagy is classified into chaperone-mediated autophagy (CMA), microautophagy and macroautophagy [41]. Macroautophagy is a ubiquitous endomembrane degradation mechanism for proteins and organelles [42]. Protein degradation of aggregated proteins in the cytosol by macroautophagy (hereafter referred to as autophagy) partakes in the degradation of protein aggregates [29,43]. Neuron-specific loss of autophagyin mice through mutations in *atg5* or *atg7* leads to adult-onset degeneration [44,45]. Hence, basal autophagy is required for neuronal maintenance in healthy neurons [44-46] (Figure 1). At low levels increased autophagy has been shown to act neuroprotectively [47,48]. Recent evidence suggests a direct role of autophagy on synaptic function. Specifically, induction of autophagy as well as basal autophagy negatively regulate neurotransmitter release and affect the presynaptic structure in dopaminergic neurons in mice [49,50]. Although the role of autophagy for synaptic maintenance was not directly investigated in these studies, it would be interesting to test if autophagic regulation of the synaptic vesicle cycle plays a direct role in synaptic maintenance. In another recent study, autophagosome biogenesis was shown at the neurite tip of neurons of the basal root ganglia [51]. The same study indicated that the primary mode of autophagosome removal from synapses is through retrograde trafficking along the axon. Furthermore, enhanced levels of presynaptic proteins, including alpha-synuclein, have been shown at synapses after cell-specific deletion of *atg7* in dopaminergic neurons [52]. Defects in endolysosomal degradation at synapses trigger the formation of large autophagosomes at synaptic terminals in *Drosophila* photoreceptors [25]. At the *Drosophila* neuromuscular junction, autophagy presynaptically regulates the number of presynaptic boutons through degradation of the E3 ubiquitin ligase highwire [53]. Ubiquitination may be part of either of the two conceptually different maintenance modes discussed above: In one mode, individual dysfunctional proteins may be recognized and marked for degradation. Alternatively, all proteins of a certain type may be ubiquitinated with a certain probability at all times, ensuring constant turnover without the need to recognize whether an individual protein has become dysfunctional. Similarly, autophagy of large protein aggregates or organelles may function in both conceptually different maintenance modes. What maintenance mode prevails at synapses is not known. In either case, a picture is emerging in which autophagy directly affects or regulates membrane trafficking at synapses and is required for the maintenance of prolonged synaptic function. However, the precise regulatory role of autophagy with respect to normal synaptic membrane trafficking, and the synaptic vesicle cycle in particular, remains unclear.

Similar to defective autophagy, several mutations in proteins that affect late endosomal or lysosomal function cause intracellular membrane accumulations and neurodegeneration (Figure 1) [22,54-56]. Defects in endosomal sorting complex required for transport (ESCRT) proteins can lead to endosomal accumulation of ubiquitinated proteins and contribute to neurodegeneration in mammalian and fly cells [57]. For example, in mammalian neurons loss of the ESCRT-III component mSnf7-2 causes retraction of dendrites and neuronal cell loss [39]. mSnf7-2 also binds to CHMP2B, an ESCRT-III subunit for which mutations have been found to cause a rare form of Frontotemporal Dementia [39,58], as discussed in the next section. Similarly, acidification defects lead to aberrant endosomal accumulations and can cause slow neuronal degeneration [59-62]. Intracompartmental acidification regulates the function of synaptic vesicles and endosomal compartments [63,64]. Although endolysosomal degradation operates in all cells, endolysosomal dysfunction often affects the nervous system before other tissues [5,22]. The dynamics of endolysosomal sorting and degradation were recently characterized at the vertebrate NMJ [65]. The authors observed synaptic ‘macroendosomes’ that contain extracellular levels of Ca^2+^ and various membrane proteins and may function as sorting endosomes, similar to those observed at motorneuron terminals in *Drosophila *[24]. Whether and when these macroendosomes are destined for local degradation, retrograde transport to the cell body or exocytosis is unclear, but all three processes seem to occur in wild type [65]. Importantly, defects in lysosomal function have also been directly linked to disrupted axonal trafficking and dystrophic defects likened to Alzheimer’s Disease [60].

In addition to ubiquitous endomembrane degradation, neurons employ specialized endolysosomal machinery. A neuron-specific branch of the endolysosomal system that predominantly functions at synapses was recently identified in *Drosophila*[25,35,61]. Loss of the neuron-specific vesicular ATPase component *v100* causes intracellular sorting and degradation at synapses [61]. Similarly, mutations in the synaptic vesicle SNARE neuronal Synaptobrevin (*n*-*syb*) cause intracellular membrane degradation defects that lead to slow adult-onset degeneration in *Drosophila*photoreceptor neurons [25]. Both *v100* and *n*-*syb*have previously been described as synaptic vesicle proteins [35,66,67]. Surprisingly, *v100* and *n*-*syb*mutant synaptic terminals in *Drosophila* are filled with endosomes, not synaptic vesicles [25]. In both mutants autophagy is initiated as a cellular response. It is not clear whether the *v100*- and *n*-*syb*-dependent neuronal ‘sort-and-degrade’ mechanism has a specificity for synaptic cargo. Alternatively, *v100* and *n*-*syb* may increase general membrane degradation. Both *v100* and *n*-*syb* have close homologs (*v0a2*-*4* and *cellubrevin*) that exert similar functions in other cell types [68,69]. The idea of a degradation mechanism with specificity for synaptic cargo is supported by the knowledge that synapses contain numerous specializations of membrane trafficking. *v100* and *n*-*syb*provide a potential molecular link between the synaptic vesicle cycle and synaptic endolysosomal sorting and degradation. A similar link has been proposed for the Rab GTPase Activating Protein (RabGAP) Skywalker [24]. In addition, a Rab11 guanine exchange factor (RabGEF) was recently shown to cause activity-dependent endolysosomal protein accumulations and adult-onset degeneration in *Drosophila* photoreceptors [70]. The recent discovery of novel synaptic endosomal Rab GTPases further suggests a role for novel, yet to be discovered, synaptic membrane trafficking machinery that functions in synaptic maintenance [71,72].

### Synaptic maintenance and Ca^2+^ homeostasis

Ca^2+^homeostasis plays a plethora of critical roles in the life of a neuron and synaptic function in particular. Ca^2+^ signaling controls early stages of neuronal differentiation and growth and the late stages of neuronal cell death [73]. Furthermore, Ca^2+^ signaling connects membrane excitability and cell biological functions of mature neurons, including synaptic plasticity underlying memory formation and retention. Tightly regulated Ca^2+^ homeostasis is a prerequisite for the precise regulation of both pre- and postsynaptic function. Presynaptic Ca^2+^ has been studied extensively [23]. However, the consequences of prolonged mild defects in presynaptic Ca^2+^ levels are less clear. Autophagosomes and lysosomes are Ca^2+^ storage compartments [11] both in the cell body and at the synapse. Intracellular Ca^2+^ directly regulates autophagy. However, in different contexts increased levels of free cytosolic calcium seem to either inhibit or promote autophagy [10]. How the tightly regulated local subcellular changes of free Ca^2+^ at synaptic terminals regulate autophagy is not known. Upon endocytosis, both synaptic vesicles and other endosomal compartments can adopt extracellular Ca^2+^ concentrations [65]. Hence, presynaptic endolysosomal compartments feature both steep H^+^ and Ca^2+^ gradients; impairments in the preservation and regulation of these gradients can lead to leakage and poisoning of synaptic function [7,61,74,75]. In particular, a specific class of lysosomal Ca^2+^ channels, the nicotinic acid-adenine dinucleotide phosaphate (NAADP)-sensitive channels, have been suggested to play a key role in the autophagic-lysosomal clearance of synaptic proteins [12]. It is therefore clear that Ca^2+^ homeostasis is required for the prolonged maintenance of presynaptic function; however, the topic still awaits dedicated investigation.

On the postsynaptic side, the role of Ca^2+^ homeostasis on longer time scales is somewhat clearer. The accepted neurophysiological correlate to learning and memory are long-term potentiation (LTP) and long-term depression (LTD). Ca^2+^ homeostasis is critical for the sustained function of LTP and LTD at the synapse. Induction of LTP, the persistent increase in synaptic strength in response to neuronal activity, is thought to be the physiological substrate of information storage in the hippocampus. In addition, induction of LTP causes an increase in spine number and spine size [76-80]. Induction of LTD, the activity-dependent reduction of synaptic transmission, results in the shrinkage of spine heads [81,82]. The precise roles of Ca^2+^ signaling in LTP and LTD has been studied extensively and is discussed elsewhere [83-85]. Here we will focus on the long-term aspects of deranged Ca^2+^ homeostasis on synaptic maintenance. In contrast to studies of synaptic plasticity, much less is known about the role of neuronal Ca^2+^ signaling in the long term at synapses.

How do small changes in Ca^2+^ homeostasis and signaling affect the properties of the synapse over of its lifetime? We would like to propose that the same Ca^2+^-dependent mechanisms which are involved in experience-evoked synaptic strengthening (LTP) and synaptic weakening (LTD)are also involved in the long-term maintenanceand elimination of synapses. Ca^2+^ influx via NMDAR and activation of Ca^2+^-dependent kinase CaMKII is a well-characterized LTP-inducing mechanism [86]. Activated CaMKII phosphorylates multiple substrates in the postsynaptic density, including scaffold protein PSD95, AMPA receptor targeting subunit stargazing and proteins involved in cytoskeleton rearrangement [84]. When compared to LTP, induction of LTD requires Ca^2+^ increases in the postsynaptic spine that are lower in amplitude but more prolonged, and often involve release of Ca^2+^ from intracellular stores in the spine [85,87]. Such slow Ca^2+^ signals cause activation of the Ca^2+^-dependent phosphatase calcineurin (CaN), which mediates dephosphorylation of synaptic proteins and weakening of the synapse. The roles of CaMKII and CaN in LTP and LTD forms of synaptic plasticity are well established [83-85]. A very similar balance (on much slower time scales) may also be necessary for synaptic maintenance and elimination. Specifically, we propose that low levels of continuous CaMKII activity are necessary to keep a “phosphorylated tone” of postsynaptic proteins. This is opposed by the continuous activity of CaN which aims to dephosphorylate postsynaptic proteins and weaken the synapse. Hence, the steady-state balance between continuous CaMKII and CaN activities may play an important role in defining life-time of individual synaptic spines. Indirect support for this hypothesis comes from the observation that disruption of the CaMKII complex with NMDAR causes persistent reduction of the synaptic strength in hippocampal synapses [88].

What are the mechanisms involved in keeping steady-state levels of synaptic CaMKII activity? One possibility is that spontaneous neurotransmitter release from the presynaptic terminal results in periodic Ca^2+^transients in the postsynaptic terminals due to intermittent activation of AMPA and NMDA receptors. Indeed, increasing recent evidence suggests the importance of spontaneous neurotransmitter release for synaptic maintenance [89]. Another alternative is Ca^2+^ influx via the neuronal store-operated Ca^2+^entry (nSOC) pathway. The molecular identity and functional role of nSOC is poorly understood [90], but it most likely includes TRPC channels and stromal interaction (STIM) molecules. The formation of excitatory spines was increased in transgenic mice that overexpresses TRPC6 channel, supporting an important role of nSOC at the synapse [91]. We recently proposed that continuous Ca^2+^ influx via nSOC may play a role in stabilizing spine structures in the central nervous system [92]. The precise contributions of spontaneous neurotransmitter release and the nSOC pathway for synaptic maintenance await further investigation.

### The maintenance problem of synapses in neurodegenerative diseases

The study of molecular neurodegeneration largely focuses on the investigation of known neurotoxic insults that include Abeta peptides in Alzheimer’s Disease (AD), polyQ proteins in Huntington Disease (HD) and Ataxias, alpha-Synuclein in Parkinson’s Disease (PD), or tau tangles in tauopathies, to name but a few [5]. All these neurotoxic insults affect the neuronal physiology, including ion homeostasis, intracellular membrane trafficking and degradation machineries. The effects on the cellular physiology can be direct due to an inherent toxic function of the disease proteins, or indirect due to a cellular response to the neurotoxic insult [5]. Hence, the often well characterized triggers of a neurodegenerative disorder and the cell biological machineries that try to keep the cell alive are closely linked. Both endomembrane degradation and Ca^2+^ homeostasis have been found to be affected in most, if not all, neurodegenerative disorders [5,7].

Despitethe differences in neurotoxic insults these disorders share numerous common features, including the observation that most of them occur in advanced age. This is particularly apparent for AD. The probability of developing AD increases exponentially with advanced age [93]. In contrast, other disorders are designated as “late-onset” but age is not as much of a risk factor as it is for AD in the sense that the probability to obtain the disease is not as much increased for, for example, the age group 70–80 compared to the prior decade. It is expected that the problems related to synaptic maintenance should manifest themselves in the aging brain and in a similar manner in early stages of AD. In this section we will therefore put particular emphasis on AD before other neurodegenerative disorders and discuss potential connections between age-related defects in synaptic maintenance and neurodegeneration.

### Impaired endomembrane degradation and synaptic degeneration

We have recently reviewed the general role of endomembrane degradation in several neurodegenerative diseases [5]. In this section we will focus on the synapse. Synaptic endomembrane degradation can be directly or indirectly affected in neurodegenerative disorders. However, some diseases are directly caused by defective membrane trafficking and in particular lysosomal function. Most prominently lysosomal storage disorders (LSDs) often affect neurons before other cell types [5,22]. Lysosomal degradation is required locally at synapses for synapse elimination and axon pruning in mouse motor neurons and the cerebellum [94]. In addition, the same study showed reduced axon removal in a mouse model for LSDs. In a *Drosophila* model for LSDs [54,95], increased oxidative stress was recently shown to create a further burden specifically for synaptic maintenance [96]. However, it is largely unclear why different lysosomal storage disorders affect varying cell types in the nervous system and elsewhere in the body differentially.

As outlined above, AD is the neurodegenerative disorder most closely linked to neuronal aging and therefore normal maintenance mechanisms. A plethora of links have been established between intracellular membrane trafficking and degradation in AD. Maybe most importantly, endolysosomal abnormalities have been observed at early preclinical stages of AD, suggesting a potential causal relationship between the cell biological defects and the subsequent pathology [97,98]. Presenilins, the catalytic subunits of the γ-secretase complex [99,100] have directly been linked to lysosomal biogenesis and function [101,102]. Numerous studies have proposed a requirement or presenlins for lysosomal function independent of its role in the γ-secretase complex, although the precise molecular mechanism remains to be determined [9,103-105]. Remarkably, presenilins seem to play a role in lysosomal Ca^2+^ storage, suggesting a potential molecular mechanism [9,105]. This role of presenilins further highlights the regulatory links between endolysosomal and Ca^2+^ homeostasis, which will be further discussed in Section 2.2. It is currently unclear, and will be interesting to see, whether familial mutations in presinilins also affect lysosomal Ca^2+^.

The amyloid precursor protein (APP) is trafficked to the plasma membrane through the secretory pathway where extracellular neurotoxic Abeta peptides are generated through beta- and gamma-secretase cleavages. The trafficking of APP may therefore determine the availability of APP to generate neurotoxic Abeta [106]. The sorting receptor sorLA binds intracellular APP and controls its plasma membrane availability. In addition, APP and secretases are also present on endosomal membranes where intracellular Abeta generation may occur [107,108]. An imbalance in the intracellular APP trafficking mechanism may be the reason why mutations in *SORL1* (the gene encoding sorLA) are associated with slow and progressive degeneration in late-onset AD [109,110]. sorLA interacts with the retromer complex, which regulates trafficking of APP and many other membrane proteins from endosomal compartments back to the golgi [110]. Loss of retromer activity causes progressive synaptic dysfunction and slow degeneration [110,111]. *SORL1* as well as sortilin, SorCS1, SorCS2 and SorCS3 are members of the *vacuolar protein sorting 10* (*vps10*) receptor family. At least sortilin has been shown to directly affect trafficking at the synapse [112]. Over time, the toxic Abeta42 variant can accumulate in late endosomal compartments and cause slow degeneration [113,114]. Abeta accumulations have also been associated with defects of the endoplasmic reticulum and mitochondria [115,116]. On the postsynaptic side, Abeta interferes with the function of the neuromodulator Reelin and ApoE receptors. Based on these findings a model has been proposed in which Abeta postsynaptically modulates both neurotransmission and synapse stability [117]. Importantly, the ApoE4 isoform of Apolipoprotein E significantly reduces the mean age-of-onset of AD. ApoE4 specifically interferes with postsynaptic glutamate receptor phosphorylation and thereby the maintenance of synaptic stability [118]. A direct link between Abeta and tau was recently shown specifically for the postsynaptic compartment: Dendritic tau may directly confer Abeta toxicity through its role in targeting the Src kinase Fyn and consequently the NMDA receptor [119]. The PAR-1 kinase was recently shown to regulate Abeta toxicity specifically postsynaptically at the *Drosophila* neuromuscular junction [120]. In addition, there is further evidence for APP/Abeta processing both on presynaptic endosomes [121] as well as in postsynaptic, somatodendritic compartments [122]. The APP cleaving enzyme 1(BACE1) predominantly colocalizes with presynaptic markers and is required for axon guidance [123]. It is not currently clear whether pre- or postsynaptic APP/Abeta processing is more critically related to AD pathology. Finally, a study by Mawuenyega et al. in 2010 highlights the more principle role of maintenance in AD: A study of central nervous system neurons in AD patients (albeit only 12 patients and 12 control) revealed normal levels of Abeta production, but impaired clearance [124]. From these studies a picture is emerging in which tightly regulated balances in membrane trafficking of APP and Abeta are required for prolonged neuronal and synaptic maintenance.

Some neurodegenerative disorders further highlight the role of impaired endomembrane degradation for synaptic maintenance. Rare cases of frontotemporal dementia as well as motor neuron diseases are caused by mutations in the ESCRT-III protein CHMP2B [58]. It is not clear why defects in CHMP2B predominantly affect the nervous system, but neuronal sensitivity to decreased endomembrane degradation as a maintenance mechanism has been suggested [40,125]. The neuropathy Charcot-Marie-Tooth 2B is caused by specific point mutations in the late endosomal small GTPase *rab7* and affects the synaptic terminals of the longest axons in the human body [126-129]. Although this disease is rare, it has attracted considerable attention due to the critical and ubiquitous requirement of *rab7* in endolysosomal degradation. How the disease mutations cause a dominant neuropathy predominantly in the nervous system is currently under investigation; several potential molecular mechanisms have been proposed based on mutant protein overexpression studies in heterologous cell lines [130-133]. However, none of these mechanism has been shown to cause axonal degeneration in motor neurons or sensory neurons *in vivo*. We are currently investigating the alternative hypothesis that partial loss of *rab7* function dominantly causes Charcot-Marie-Tooth 2B and thereby reveals dosage-dependent neuronal sensitity to reduced endolysosomal degradation.

Niemann-Pick disease type C is caused by mutations in the endolysosomal membrane protein NPC1 and characterized by cholesterol accumulation in late endosomal or lysosomal compartments. Synapses of both excitatory and inhibitory neurons deficient for NPC1 develop normally, but exhibit progressive functional defects [134]. These findings suggest a requirement for correct Cholesterol homeostasis during prolonged synaptic function, which is reviewed elsewhere [134,135].

Finally, autophagy has been linkedto synaptic maintenance in neurodegenerative disorders characterized by tau or polyQ accumulations [136]. Decreased autophagy results in increased tau aggregation and toxicity [137,138]. Inhibition of tau phosphorylation or genetic deletion of tau can partially suppress neurodegeneration caused by autophagy suppression [139]. Interestingly, the same study showed that this suppression did not correlate with any effect on inclusion formation. Furthermore, it has been proposed that autophagy is directly affected by Huntingtin-polyQ [140]. Disrupted autophagy has further been shown to lead to dopaminergic axon degeneration and presynaptic alpha-synuclein and LRRK2 accumulation [52]. Tau-mediated synaptic toxicity of Abeta is regulated by ubiquitination and degradation and directly affects synaptic morphology and function [120]. A proteasomal response to tau accumulations can trigger autophagy [36]. Disruption of the autophagosomal/lysosomal system and *vps41*-mediated neuroprotection has been shown in PD [141] and both retromer and lysosomal defects have been linked to increased PD risk [142]. Finally, a mouse knock-in model for the ataxia SCA6, which is caused by mutations in a voltage-gated Calcium channel, revealed severe lysosomal defects as part of its pathogenic mechanism [13]. This example further highlights the Ca^2+^ and endomembrane systems. All these examples highlight how neurotoxic proteins can exacerbate a maintenance defect through impaired endomembrane degradation.

### Synaptic degeneration and Ca^2+^ homeostasis

**Figure 2:**
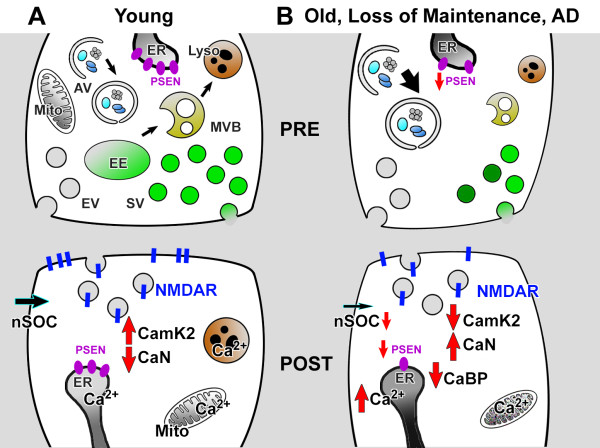
**Ca**^**2+**^** Signaling and synaptic maintenance. A.** Synaptic maintenance requires continuous trans-synaptic signaling at excitatory synapse. Spontaneous release of glutamate triggers activation of NMDA receptors (NMDAR) and Ca^2+^ elevation in the postsynaptic terminal. Low levels of Ca^2+^ in postsynaptic ER causes Ca^2+^ influx via the nSOC pathway. These Ca^2+^ signals continuously stimulate activity of CaMKII, which is necessary for maintenance of synaptic spine structure. **B.** Similar Ca^2+^ signaling defects are observed at the presynaptic terminal and postsynaptic spines in synapses that old, exhibit premature loss of maintenance machinery or in Alzheimer’s Disease (AD). The frequency of spontaneous glutamate release is diminished in both aging and AD neurons. Ca^2+^ influx via NMDARs is reduced. Mitochondria are depolarized and less effective in Ca^2+^ uptake. The levels of cytosolic CaBPs are reduced. The ER Ca^2+^ levels are increased and synaptic nSOC is diminished. As a result of these changes the activity of CaMKII at the synapse is reduced and activity of CaN is elevated, leading to weakening and destabilization of the synapses in aging and AD neurons by promoting “LTD-like” signaling pathways. Similar, but more severe, processes are observed in PS-FAD neurons. Red arrows indicate increased or decreased activity. ER - endoplasmic reticulum; AV - autophagic vacuole; Mito - Mitochondrium; EE - early endosome; EV - endocytic vesicle; SV - synaptic vesicle; nSOC - neuronal store-operated Ca^2+^ entry; NMDAR - NMDA receptor; CamK2 - Ca/CaM Kinase 2; CaN - Calcineurin; CaBP - Calcium binding protein; PSEN - Presenilin.

In Section 1.2 we proposed that synaptic maintenance requires a balance between “LTP-like” (CaMKII-mediated) and “LTD-like” (CaN-mediated) synaptic signaling pathways. We would like to discuss the idea that this balanceis shifted towards “LTD-like” pathways in aging neurons. This may leads to late-onset synaptic loss and age-related cognitive decline (Figure 2). There is a considerable amount of indirect evidence in support of this hypothesis. Studies of synaptic plasticity revealed the shift in susceptibility to LTD in aging neurons [143]. This shift is due to increased contribution of intracellular Ca^2+^ stores [144] and is paralleled by the shift of the balance from kinases to phosphatases in the synapse [145]. Multiple studies of aging neurons pointed to increased Ca^2+^ release from intracellular stores via InsP_3_R and RyanR, increased Ca^2+^ influx via L-type VGCC, and reduced contribution of NMDAR-mediated Ca^2+^ influx [8,146,147]. All these changes are expected to shift the balance from CaMKII-mediated “synaptic maintenance” to CaN-mediated “synaptic weakening” (Figure 2).

There are several potential reasons for these Ca^2+^ signaling changes in aging neurons. One factor is reduced cytosolic Ca^2+^ buffering capacity. The levels of neuronal cytosolic Ca^2+^ -binding proteins (CaBPs) are reduced in aging neurons [8,147]. A second factor is reduced mitochondrial function due to cumulative oxidative damage to mitochondria. The mitochondria from aged neurons are depolarized and less efficient in handling high Ca^2+^ loads [8,147]. It is likely that reduced levels of CaBPs and reduced mitochondrial Ca^2+^ uptake capacity force ER Ca^2+^ stores to play a larger role in Ca^2+^ handling in aging neurons (Figure 2). We hypothesize that increased levels of ER Ca^2+^ in aging neurons result in a downregulation of nSOC pathway function and reduced steady-state levels of CaMKII activity in the spines (Figure 2). We also propose that increased ER Ca^2+^ levels facilitate the activation of CaN (Figure 2). Indeed, CaN activity is enhanced in aging neurons and plays an important role in increased LTD [148,149].

Similar ideas may explain synaptic loss in AD. In a recent review article we hypothesized that abnormal neuronal Ca^2+^ signaling may play an important role in destabilizing mature synaptic spines in AD [92]. Elevated levels of CaN activity has been observed in AD human brains [150-152] and dysregulated phosphorylation of CaMKII was reported for MCI and AD human brains [153]. The importance of CaN was further highlighted by multiple studies in mouse models of AD. The morphological alterations in neurites could be reduced by treatment with the CaN inhibitor FK-506 *in vitro* and *in vivo* in AD mouse models [154-157] and inhibition of CaN resulted in memory deficit rescue in an AD mouse model [158]. All these results indicate a shift in the balance from CaMKII towards CaN in AD synapses. On a mechanistic level these effects are usually interpreted as the result of synaptotoxic action of oligomeric Aβ42 [159]. However, it is possible that the balance between CaMKII and CaN activity in the synapse can be tilted as a result of increased ER Ca^2+^ levels and resulting changes in intracellular Ca^2+^ homeostasis. The strongest evidence in support of this idea comes from the analysis of familial AD (FAD)-causing mutations in presenilins (*PSEN1* and *PSEN2 *genes) [160]. Presenilins act as catalytic subunits of the γ-secretase complex which cleaves type-1 transmembrane proteins, including Notch [99,100]. The majority of genetically-linked FAD is caused by missense mutations in the *PSEN1* and *PSEN2 *genes. Many of the PS FAD mutations result in enhanced Ca^2+^ release via inositol 1,4,5-trisphosphate receptors (InsP_3_R) and ryanodine receptors (RyanR) [161-164]. To explain these findings, we previously demonstrated that in addition to acting as the catalytic component of the γ-secretase complex, presenilins also function as passive ER Ca^2+^ leak channels, a function disrupted by many FAD mutations. We reasoned that the loss of ER Ca^2+^ leak function of presenilins leads to increased endoplasmic reticulum (ER) Ca^2+^ levels and enhanced ER Ca^2+^ release in PS-FAD cells [160,165-167]. Independent experimental support for the leak function of presenilin is accumulating [168,169]. A large hole that traverses through the entire protein was observed in the recent high resolution crystal structure of the archaeal presenilin homologue PSH1, which could either indicate a cavity for water access or underlie the ion channel properties [170].

Many FAD mutations in presenilins disrupt the ER Ca^2+^ leak function and result in elevated ER Ca^2+^ levels [160,165-167,171] and impaired store-operated Ca^2+^ entry [166,171,172]. As discussed above, increased ER Ca^2+^ levels are one of the signature features of aging neurons [146]. Thus, studies with PS-FAD mutant neurons provide an opportunity to investigate alterations in Ca^2+^-dependent synaptic signaling which would typically only occur in aging neurons. Consistent with this hypothesis, an altered balance between the induction of LTP and LTD at synapses was indeed observed in experiments with PS1-FAD neurons [173,174]. These differences were uncovered following inhibition of RyanR-mediated Ca^2+^ release by dantrolene, suggesting that intracellular Ca^2+^ stores exert large effect on synaptic plasticity in PS1-FAD neurons but not in wild type neurons. These findings further suggest that synaptic transmission in PS1-FAD neurons operates under a ‘shifted homeostatic state’ [173,174]. In addition to the changes in postsynaptic ER Ca^2+^ signaling described above, FAD mutations in presenilins also exert effects on presynaptic ER Ca^2+^ handling and neurotransmitter release [175,176]. FAD mutations in presenilins also influence homeostatic synaptic scaling [177]. Thus, it is possible that FAD mutations in presenilins affect synaptic maintenance from the presynaptic side as well, for example by lowering the frequency of spontaneous neurotransmitter release and interfering with homeostatic trans-synaptic mechanisms (Figure 2).

Similar to AD, synaptic pathology has been implicated in many other neurodegenerative disorders. For example, there is extensive evidence for dysregulated cortico-striatal synapses at early stages of Huntington’s disease (HD). It appears that synaptic changes in HD result mainly from changes in cell biological and Ca^2+^ signaling mechanisms induced by mutant Huntingtin protein [178-181]. It is however possible that age-related synaptic maintenance defects outlined in this review contribute to the vulnerability of synapses to other toxic insults, such as mutant Huntingtin-polyQ protein. It is therefore possible that a therapeutic strategy that favors synaptic maintenance (such as for example selective activation of endomembrane degradation or inhibition of CaN) may proof beneficial in these disorders as well by making synapses more resistant to further toxic insults.

## Conclusion

In this review we attempted to highlight the importance of synaptic maintenance for neuronal health and disease. In particular, we focused on endomembrane degradation and Ca^2+^ signaling and the cross-regulation. Mild dysregulation or defects in either of these processes are likely to lead to slow synaptic degeneration. Aberrant endomembrane degradation and Ca^2+^ signaling may thus contribute to synaptic loss and age-related cognitive decline. The neurodegenerative disorder that most closely resembles the loss of synaptic maintenance phenotypes is AD. However, defects in synaptic maintenance may also contribute to synaptic vulnerability in other neurodegenerative disorders. Many of our conclusionsare inferred from short-term experiments, but the experimental tools to test these proposals in long-term experiments are becoming increasingly available. Testing these ideas may help to understand the cell biological mechanisms underlying late-onset synaptic degeneration and facilitate the development of novel therapeutic agents.

## Competing interests

The authors declare that they have no competing interests.

## Authors’ contributions

IB and PRH wrote this review. All authors read and approved the final manuscript.
